# Supplementary material for: The Diversity of Wolbachia and Other Bacterial Symbionts in Spodoptera frugiperda
Source: Insects. 2024 Mar 22;15(4):217. doi: 10.3390/insects15040217 (PMC11050099; doi:10.3390/insects15040217)
Supplement: Supplementary file 1 [file insects-15-00217-s001.zip › insects-2876447-supplementary.pdf]

Table S1. The reference sequences of the *Wolbachia wsp* genes used in the phylogenetic analysis.

Supergroup	Subgroup	Host	GenBank accession number
A	Dro	*Trichogramma drosophilae*	AF071910
	Mel	*Drosophila melanogaster*	AF020065
		Drosophila melanogaster	AF020063
		Drosophila melanogaster	AF020072
		Drosophila melanogaster	AF020064
		Drosophila melanogaster	AF020066
		Drosophila simulans	AF020067
	AlbA	*Aedes albopictus*	AF020058
	Kue	*Trichogramma kaykai*	AF071912
		Ephestia kuehniella	AF071911
		Trichogramma bourarachae	AF071913
	Riv	*Drosophila simulans*	AF020070
		Drosophila auraria	AF020062
	Mors	*Glossina morsitans*	AF020079
		Nasonia vitripennis	AF020081
		Glossina morsitans centralis	AF020078
	Uni	*Muscidifurax uniraptor*	AF020071
	Haw	*Drosophila simulans*	AF020068
		Drosophila sechellia	AF020073
		Drosophila cautella	AF020075
	Pap	*Phlebotomus papatasi*	AF020082
	Aus	*Glossina austeni*	AF020077
B	For	*Bemisia tabaci*	MK157080
		Encarsia formosa	AF071918
	CauB	*Cadra cautella*	AF020076
		Tagosodes orizicolus	AF020085
	Vul	*Armadillidum vulgare*	AF071917
	Pip	*Drosophila simulans*	AF020069
		Drosophila simulans	AF020074
		Culex quinquefasciatus	AF020060
		Culex pipiens	AF020061
	Div	*Apoanagyrus diversicornis*	AF071916
	Kay	*Trichogramma kaykai*	AF071924
		Trichogramma nubilate	AF071926
		Trichogramma deion	AF071925
		Trichogramma kaykai	AF071927
	Sib	*Trichogramma sibericum*	AF071923
	Dei	*Trichogramma deion*	AF020084
	Con	*Laodelphax striatellus*	AF020080
		Tribolium confusum	AF020083
D	——	*Brugia pahangi*	AY527207
